# Testing configurations of attractive toxic sugar bait (ATSB) stations in Mali, West Africa, for improving the control of malaria parasite transmission by vector mosquitoes and minimizing their effect on non-target insects

**DOI:** 10.1186/s12936-021-03704-3

**Published:** 2021-04-14

**Authors:** Rabiatou A. Diarra, Mohamed M. Traore, Amy Junnila, Sekou F. Traore, Seydou Doumbia, Edita E. Revay, Vasiliy D. Kravchenko, Yosef Schlein, Kristopher L. Arheart, Petrányi Gergely, Axel Hausmann, Robert Beck, Rui-De Xue, Alex M. Prozorov, Aboubakr S. Kone, Silas Majambere, John Vontas, John C. Beier, Günter C. Müller

**Affiliations:** 1 Malaria Research and Training Center, Faculty of Medicine, Pharmacy and Odonto-Stomatology, University of Sciences, Techniques and Technology of Bamako, BP 1805 Bamako, Mali; 2 Department of Zoology, Tel Aviv University, 61500 Tel Aviv, Israel; 3 Department of Microbiology and Molecular Genetics, IMRIC, Kuvin Centre for the Study of Infectious and Tropical Diseases, Faculty of Medicine, Hebrew University, Jerusalem, Israel; 4 Department of Public Health Sciences, Miller School of Medicine, University of Miami, Miami, FL USA; 5 SNSB-Zoologische Staatssammlung München, 81247 München, Germany; 6 Anastasia Mosquito Control District, 120 EOC, St. Augustine, FL 32092 USA; 7 Pan-African Mosquito Control Association, P.O. Box 54840-00200, Nairobi, Kenya; 8 Institute of Molecular Biology and Biotechnology, Foundation for Research and Technology-Hellas, 70013 Heraklion, Greece; 9 Pesticide Science Laboratory, Department of Crop Science, Agricultural University of Athens, 11855 Athens, Greece

**Keywords:** Non-target organisms (NTOs), *Anopheles gambiae* s.l., ATSB, ASB, Diptera, Lepidoptera, Hymenoptera

## Abstract

**Background:**

Application methods of |Attractive Toxic Sugar Baits (ATSB) need to be improved for wide-scale use, and effects on non-target organisms (NTOs) must be assessed. The goals of this study were to determine, at the village level, the effect of different configurations of bait stations to (1) achieve < 25% *Anopheles* mosquito vector daily feeding rate for both males and females and (2) minimize the effect on non-target organisms.

**Methods:**

Dye was added to Attractive Sugar Bait Stations (without toxin) to mark mosquitoes feeding on the baits, and CDC UV light traps were used to monitor for marked mosquitoes. An array of different traps were used to catch dye marked NTOs, indicating feeding on the ASB. Stations were hung on homes (1, 2, or 3 per home to optimize density) at different heights (1.0 m or 1.8 m above the ground). Eight villages were chosen as for the experiments.

**Results:**

The use of one ASB station per house did not mark enough mosquitoes. Use of two and three stations per house gave feeding rates above the 25% goal. There was no statistical difference in the percentage of marked mosquitoes between two and three stations, however, the catches using two and three bait stations were both significantly higher than using one. There was no difference in *An. gambiae* s.l. feeding when stations were hung at 1.0 and 1.8 m. At 1.8 m stations sustained less accidental damage. ASB stations 1.8 m above ground were fed on by three of seven monitored insect orders. The monitored orders were: Hymenoptera, Lepidoptera, Coleoptera, Diptera, Hemiptera, Neuroptera and Orthoptera. Using one or two stations significantly reduced percentage of bait-fed NTOs compared to three stations which had the highest feeding rates. Percentages were as follows: 6.84 ± 2.03% Brachycera followed by wasps (Hymenoptera: Vespidae) 5.32 ± 2.27%, and Rhopalocera 2.22 ± 1.79%. Hanging the optimal number of stations per house for catching mosquitoes (two) at 1.8 m above ground, limited the groups of non-targets to Brachycera, Chironomidae, Noctuoidea, Rhopalocera, parasitic wasps and wasps (Hymenoptera). Feeding at 1.8 m only occurred when stations were damaged.

**Conclusions:**

The goal of marking quarter of the total *Anopheles* population per day was obtained using 2 bait stations at 1.8 m height above the ground. This configuration also had minimal effects on non-target insects.

## Background

The search for methods to enhance the present arsenal of mosquito control continues as malaria remains a devastating disease [1]. A new method, Attractive Toxic Sugar Baits (ATSB), has been successfully used against populations of *Anopheles* as well as several other mosquito species [2, 3, 5–7]. Sugars are a staple diet of mosquitoes and the ATSB imitates the mosquito attraction to natural sources by olfactory cues and offers food that can be “spiked” with various toxins such as carbamates, pyrethroids, neonicotinoids, spinosyns, borates, and biopesticides [2–7]. Toxic sugar baits have been used as a foliar spray on green vegetation and blossoms [2, 3] and also as an ATSB which can be hung on the external walls of houses or inside cisterns or drains [4].

The success of ATSB spray against mosquitoes, particularly on flowering vegetation [2], raised concerns as to whether ATSB, could affect populations of non-target organisms such as bees and butterflies. It was found that an unacceptable variety of species were stained when non-toxic Attractive Sugar Bait (ASB) was used to dye-mark non-target insects in simulated kill experiments [8, 9]. The solution to spray only green vegetation with ATSB [8–10] and to issue instructions with commercial ATSB formulations to only spray up to 5% of the green vegetation. The same concern with early bait station prototypes led to the suggestion that a physical barrier, such as a fine wire mesh cage, be added to the outside to prevent entry of the larger non-targets [8].

In this study, a new prototype ATSB station suitable for wide-scale use was tested and the goals were 1) to identify effective outdoor village-level configurations of bait stations needed to achieve a minimum average dye-marked vector prevalence rate of over 25% for both males and females, and 2) to determine bait station configurations that minimize the impact on non-target organisms (NTOs). The durability and efficacy of the bait over time was also evaluated.

## Methods

### Villages

Experiments were carried out in the following villages, located in the Koulikoro Province of Mali, West Africa: Tiko (12.13444, − 8.396860), Balala (11.96599, − 8.468310), Niaganabougou (12.15466, − 8.308260), Sambadani (12.14454, − 8.316880), Korea (12.04576, − 8.399230), Krekrelo (11.98836, − 8.551460), Cissebougou (12.09628, − 8.372850), and Trekorou (12.068577, − 8.314414). The villages, accessible by car, were within 10 km of the Niger River, and had high densities of *Anopheles gambiae *sensu lato (s.l.).

### Bait station construction and ASB Composition

ASB consisted of the following: ~ 22% (w/w) date syrup as the attractant component, 77% (w/w) brown sugar ("Nature Sugar" brown, Louis Dreyfus, Israel) as the feeding stimulus, 10% (w/w) of a proprietary mixture of slow-release substances, (BaitStab ™, Westham Innovations Ltd., Tel Aviv, Israel) which is a proprietary preservative component, and 0.5% (w/w) orange food dye (Carmoisine E122, Stern, Natanya, Israel). A similarly prepared solution with green food dye (Tartrazine 19,140, Stern, Natanya, Israel) instead of orange was used for the 1.0 m height experiment. Bait stations were constructed using a white, rectangular plastic frame, 24 cm w by 36 cm l, with the ASB inside a proprietary, permeable, black SEBS (Styrene Ethylene Butylene Styrene) membrane (100 g of ASB inside 16 cells of membrane Westham Innovations LTD, Tel Aviv, Israel).

### Optimal bait station number on external house walls

Monitoring in each of eight villages was carried out with ten CDC UV (John W. Hock Co., Gainesville, FL, USA) light traps, set in the centre of the village, 5 m from houses and at least 10 m away from each other in a rough grid pattern for eighteen nights in 2016. ASB station coverage consisted of one, two or three bait station(s) with dye on every house in the village; if more than one bait station was used, they were situated on opposite walls at a height of 1.8 m. On August 17, 24, 30, a single ASB station was hung at 1.8 m above the ground on each house as well as on September 04, 10, 16, and 27. Two bait stations were hung per house on August 19 and 26, and on September 02, 05, 12, and 20. three bait stations were hung per house on August 21 and 29 as well as on September 09, 13 and 25. CDC Traps were set at 18:00 h and emptied at 06:00 h the following day.

### Effect of bait station height

Monitoring with CDC Traps from 18:00 h to 06:00 h as described above was carried out on nine nights: July 25, 27, 29, August 05, 09, 08, 11, 12, and 15 with ASBs deployed in which 2 ASBs were situated on the same wall at 1.0 m and 1.8 m above the ground on two opposing walls of each house in the village. ASBs at 1.0 m contained green stain while the stain in the ASB positioned 1.8 m was orange.

### Performance of fresh versus aged baits

ASB coverage consisted of 2 bait stations on every house, side by side, 2 m apart, both 1.8 m above the ground, one fresh, the other aged under field conditions (by hanging outside near the study area) for 6 months each with differently coloured bait (orange and green). The trials ran for six days and nights, during which feeding rates of *An. gambiae s.l.* were evaluated and dates included October 1, 2, 6, 8, 11 and 13. Mosquitoes were collected in each village with 10 CDC-UV light traps set in the centre of the village, 5 m from houses but at least 10 m away from each other. Traps were set at 18:00 h and emptied at 06:00 h the following day.

### NTO monitoring

NTOs were monitored after the ASB deployment in each village with 50 yellow plates (yellow disposable plastic plates 25-cm diameter filled with water and a drop of Triton X-100 as detergent), four Malaise traps (6 m; John W. Hock Co., Gainesville, FL, USA), two ultraviolet light traps (generator powered 250 W ML light bulb mounted in front of a white 2 m × 5 m white linen sheet), six ultraviolet tray traps [13], 50 pitfall traps (500 ml plastic cups buried to the rim in the ground, baited with 10 ml vinegar), sweep nets (BioQuip, Rancho Dominguez, CA, USA) (operated by two collectors), and aerial hand nets (BioQuip, Rancho Dominguez, CA, USA). Collected insects were stored in paper envelopes and petri dishes at − 20 °C before being processed for identification.

Stained specimens were separated from unstained specimens in the catches, stained specimens were pooled and identified with assistance from experts from the Entomological Department at the ZSM Natural History Museum (Zoologische Staatssammlung München). ASB feeding evaluation focused on seven of the most common orders. Feeding was determined by dissecting and examining guts for food dye under a 10X dissecting microscope. The insect orders were: Hymenoptera [ants (Formicidae) bees (Apidae), and wasps (Vespidae)], Lepidoptera (Rhopalocera, Bombyces, Geometroidea, Noctuoidea, Sphingidae, Pyraloidea), Coleoptera (Carabidae, Tenebrionidae, Scarabaeidae, Cerambycidae, and Chrysomelidae), Diptera (Brachycera, Chironomidae), Hemiptera (Cicadomorpha and Heteroptera), Neuroptera (Myrmeleontiformia) and Orthoptera (Caelifera and Ensifera).

### Statistical methods

#### Bait station number analysis

A generalized linear mixed model to compare the effect of the number of bait stations on the number of mosquitoes that fed on the bait and were caught in the traps. Female and male mosquitos were analyzed analyzed separately. The fixed effects in the model were village and number of bait stations which was a repeated measure. The random term was traps nested within villages that formed the error term for the repeated measure of number of bait stations. A compound symmetric covariance matrix was used to represent the correlated data structure. The total number of males and females trapped were included as an offset to produce model mean percent, standard error, and 95% confidence bounds. P-values for comparisons are given for the mean percent of mosquitoes that fed on the bait among the number of bait stations.

#### Bait station height analysis

This analysis compared the number of mosquitoes that fed on the bait and were caught by traps at a height of 1.0 m and 1.8 m. A generalized linear mixed model for Poisson outcome: the number of mosquitoes caught. Female and male mosquitoes were analyzed separately. The fixed effects in the model were village and trap height which was a repeated measure. The random term was traps nested within villages that formed the error term for the repeated measure of height. A compound symmetric covariance matrix was used to represent the correlated data structure. The total number of males and females trapped was used as an offset to produce model mean percent, standard error, and 95% confidence bounds. P-values are presented for the comparison of the mean percent of mosquitoes that fed on the bait at each trap height.

#### Aged versus fresh baits

A generalized linear mixed model to compare the effect of bait age on the relative number of mosquitoes that fed on fresh and 6-month old ASB stations. Female and male mosquitoes were analyzed separately. The fixed effects in the model were village and bait age which was a repeated measure. The random term was traps nested within villages that formed the error term for the repeated measure of number of bait stations. A compound symmetric covariance matrix was used to represent the correlated data structure. The total number of males and females trapped was included as an offset to produce model mean percent, standard error, and 95% confidence intervals. P-values were presented for comparisons of the mean percent of mosquitoes that fed on the bait between the two ages of the bait.

#### Effect of bait station number on non-targets

A generalized linear mixed model was used to compare the effect of the number of bait stations on the number of insects that fed on the bait and were caught in the traps. The fixed effects in the model were village, type of insect, number of bait stations, and the interaction of insect type with number of bait stations. Type of insect and number of bait stations were repeated measures. The random term was traps nested within villages that formed the error term for the repeated measures. A compound symmetric covariance matrix was used to represent the correlated data structure. The total number of insects trapped was used as an offset to produce model mean percent, standard error, and 95% confidence bounds. For each insect group, p-values were presented for comparisons of the mean percent of insects that had fed on the bait, between number of bait stations.

#### Effect of height on non-targets

This analysis compared the number of insects that fed on the bait at a height of 1.0 m and 1.8 m. Separate analyses were performed for the different heights because the type of insects caught were different, depending on the height of the bait station. A generalized linear mixed model was used for Poisson outcome: the number of insects that fed on the bait. The fixed effects in the model were village and type of insect which was a repeated measure. The random term was traps nested within villages that formed the error term for the repeated measure of type of insect. A compound symmetric covariance matrix was used to represent the correlated data structure. The total number of insects trapped was included as an offset to produce model mean percent, standard error, and 95% confidence bounds. The p-value is presented for the comparison among the mean percent of each type of insect that had fed on the bait.

Analyses were carried out using SAS 9.4 (SAS Institute, Inc.; Cary, NC). The two-tailed alpha level was used to determine the statistical significance of all statistical tests.

## Results

### Choice of bait station number on the external walls of huts

In the seven trials with a single bait station 23.1% of the females and 14.6% of the males were dye marked by ASB. During the six trials using two bait stations per building, the percentage of dye marked females rose to 33.75% of the females and 30.62% of the males. Three bait stations increased the percentage of the labelled *An. gambiae s.l.* only slightly to 39.11% females (330/ 826) and 32.77% males.

Using one station did not achieve male or female average daily vector feeding rates of 25% (Table 1). Though there was a significant difference between 2 and 3 bait stations for males and females, the differences only amounted to 2.15 and 5.36%, respectively. Therefore, in subsequent experiments, it was determined that two bait stations hung from each building was sufficient to achieve target daily feeding rate of 25%.

**Table 1:** Mean number of *An. gambiae s.l.* caught per night per village ± SE and mean percentage of catch labelled by the ASB using one, two and three stations

		Females				Males		
	p-value	Diff	95% CI UL	95% CI LL	p-value	Diff	95% CI UL	95% CI LL
1.8 vs 1.0	0.5170	3.44	11.18	0.00	0.9934	0.34	7.40	0.00

*UL* upper limit, *LL *lower limit, *Diff*. Difference between means

### Choice of bait station height on the external walls of huts

During nine trials, a total of 1,114 female and 665 male *An. gambiae s.l.* were collected. These included 14.22% of the females and 12.06% of the males that fed from the stations 1.8 m above the ground, and 11.86% of the females and 12.20% of the males that fed on the stations 1.0 m above the ground (Table 2). There was no statistical difference between the number of males *versus* females stained by bait stations hung at 1.0 m (p = 0.3755; 95% CI 0.00 to 11.96) or at 1.8 m (p = 0.518 95% CI 0.2605 to 15.74). There was also no difference between females labelled at 1.0 m *versus* those labelled at 1.8 m (p = 0.5170; 95% CI 0.00 to 11.18) or males labelled at 1.0 m *versus* labelling at a height of 1.8 m (p = 0.9934; 95% CI − 8.08 to 7.40). The remainder of the caught mosquitoes (49.66%) were not labelled.

**Table 2:** Mean number of *An. gambiae* s.l. per night per village ± SE and mean percentage of catch stained by the ASB stations at 1.0 and 1.8 m

		Females				Males		
	p-value	Diff	95% CI UL	95% CI LL	p-value	Diff	95% CI UL	95% CI LL
1 vs 2 station	0.0044	36.71	63.23	10.20	0.8350	8.00	34.52	0
1 vs 3 station	0.0002	56.50	87.59	25.41	0.5683	14.61	45.70	0
2 vs 3 station	0.3141	19.79	50.88	0.00	0.9336	6.61	37.70	0

*UL* upper limit, *LL *lower limit, * Diff*. Difference between means

Thirty-four (out of 1,409) of the lower stations were damaged accidentally by humans and animals walking by, resulting in insect invasion (which means insects found inside of a bait station). Most observed insect invasions (29 out of 34) were by ants, usually on one of the damaged stations. Social wasps invaded one of the higher stations while bees invaded one of the lower damaged stations.

The mean difference in the numbers of marked *An. gambiae s.l.* that had been labelled by ASB at a height of 1.8 m *versus* 1.0 m were not significantly different for either females (P = 0.5170) or males (P = 0.9934).

### Performance of fresh versus aged baits

In the experiment comparing fresh and aged ASB bait stations, 796 female and 251 male *Anopheles* were collected. From those, 16.66.% and 16.19% of the females fed from fresh and aged bait stations, respectively. Of the males, 15.28% and 15.12% fed on fresh *vs* aged bait, respectively. There were no statistical differences between males and females feeding on fresh *vs.* aged baits (Table 3).

**Table 3:** Mean number of *An. gambiae s.l.* caught per night per village ± SE and mean percentage of the catch marked by the ASB using fresh and aged bait stations

		Females				Males		
	p-value	Diff	95% CI UL	95% CI LL	p-value	Diff	95% CI UL	95% CI LL
Fresh vs Aged	0.4239	0.47	3.35	0.00	0.8158	0.67	3.46	0.00

*UL* upper limit, *LL *lower limit, * Diff*. Difference between means

The mean difference in the numbers of marked *An. gambiae *s.l. that had been labelled by ASB when fresh and aged bait stations were used was not significant for females (p = 0.4239) or males (p = 0.8158).

### Correlation between the number of ASB stations and the presence of the dye marker in non-target insect species.

To determine the impact of number of stations on non-targets insects, 57,902 captured insects from all villages were pooled and dissected. The dye marker was observed in 162 (0.3%) specimens belonging to different insect groups as shown in Table 4.

**Table 4:** Species composition among dye-marked specimens

Group	Mean%	SE	LL 95%	UL 95%
Formicidae (Ants)	1.39	0.79	0.45	4.26
Bombyces complex	0.29	0.03	0.03	2.60
Brachycera	5.73	1.61	2.34	14.02
Caelifera	0.57	0.06	0.06	5.08
Carabidae	0.28	0.03	0.03	2.54
Cerambycidae	1.04	0.82	0.12	9.22
Chironomidae	1.60	0.89	0.54	4.75
Cicadomorpha	0.58	0.06	0.07	5.19
Ensifera	1.23	0.70	0.23	6.59
Geometroidea	0.28	0.02	0.05	1.52
Heteroptera	0.93	0.37	0.21	4.05
Apidae (Honey bees)	1.74	0.94	0.60	5.04
Myrmeleontiformia	3.02	1.59	0.56	16.27
Noctuoidea	1.00	0.56	0.33	3.02
Vespidae (Parasitic wasps)	1.19	0.82	0.31	4.65
Pyraloidea	0.66	0.24	0.17	2.52
Rhopalocera	1.74	0.91	0.51	5.97
Coleoptera: Scarabaeidae (beetles)	0.83	0.36	0.19	3.65
Coleoptera: ("other beetles")	0.62	0.38	0.19	2.07
Sphingidae	1.18	1.01	0.22	6.37
Vespidae (Wasps)	4.91	1.33	1.93	12.48
Wild Bees (Apidae)	0.48	0.03	0.13	1.73

Mean percentage per night per village of dye marked specimens representing different insect orders in the combined catches of one, two and three stations (N = 162 dye marked specimens)

LL—95% confidence interval lower limit, UL—95% confidence interval upper limit

The combined catches caught a wide variety of stained insects; 22 groups belonging to seven orders were stained (Table 4). The highest number of stained specimens were from Brachycera (5.73 ± 1.61SE), Vespidae (4.91 ± 1.33SE), and Myrmeleontiformia (3.02 ± 1.59SE). Except for Heteroptera, the number of dye-marked specimens was in proportion to the number of bait stations hung (Table 5) as determined by the average catches of the various NTO trapping methods.

**Table 5:** Comparison of the percentage of stained NTO’s per night per village by CDC-UV traps Only species collected at all three trapping densities could be compared

Group	% with 1 Station				% with 2 Stations				% with 3 Stations				Comparison P-values		
	Mean %	± SE	LL 95%	UL 95%	Mean %	± SE	LL 95%	UL 95%	Mean %	± SE	LL 95%	UL 95%	1 vs. 2*	1 vs. 3*	2 vs. 3*
Formicidae (Ants)	1.04	0.97	0.16	6.64	1.11	0.90	0.22	5.54	11.94	3.57	0.87	16.39	0.956	0.139	0.132
Brachycera	3.89	1.58	1.04	14.51	6.84	2.03	2.12	22.09	16.72	3.12	1.35	20.79	0.510	0.319	0.530
Heteroptera	1.04	1.21	0.10	10.64	0.74	0.85	0.08	7.26	4.78	1.61	0.20	11.33	0.836	0.446	0.348
Noctuoidea	0.35	0.04	0.03	3.55	1.48	0.83	0.37	5.93	4.78	1.53	0.32	7.24	0.282	0.153	0.452
Rhopalocera	1.04	1.21	0.10	10.64	2.22	1.79	0.44	11.07	9.55	3.63	0.56	16.30	0.589	0.237	0.380
Vespidae (Wasps)	3.32	1.34	0.82	13.47	5.32	2.27	1.57	18.02	17.20	2.07	1.34	22.06	0.602	0.272	0.418
Wild Bees (Apidae)	0.27	0.30	0.03	2.48	0.31	0.04	0.03	3.07	4.78	1.53	0.32	7.24	0.922	0.110	0.133

^*^Number of stations

LL—95% confidence interval lower limit, UL—95% confidence interval upper limit

### Relationship between height of placement of ASB stations and the presence of dye marker in non-target insect species

The availability of bait stations at a height of 1.0 m and 1.8 m to non-target insects was compared. A total number of 34,401 specimens was captured and dissected, 446 of which had dye labelled by the colour of low hanging stations, and 32 specimens were marked by the dye that was within the high hanging stations (Table 6). In total, 1.39% of the collected specimens were dye labelled (1.23% from exposure to ASBs hung at 1.0 m and 0.09% of specimens were exposed to dye at ASB stations hung at 1.8 m).

**Table 6:** Species composition of dye marked insects caught at 1.0 m (N = 446) and 1.8 m (N = 32). Standard Error (SE) and the lower and upper limits at the 95% confidence interval (CI) are shown

Group	Mean	SE	LL 95%	UL 95%
Formicidae (Ants)	7.73	3.81	2.92	20.42
Bombyces complex	1.57	1.16	0.36	6.78
Brachycera	15.07	4.92	6.09	37.28
Caelifera	1.06	1.17	0.12	9.32
Carabidae	0.53	0.38	0.06	4.66
Cerambycidae	3.67	3.10	0.70	19.44
Chironomidae	9.75	3.72	3.75	25.36
Chrysomelidae	0.53	0.28	0.06	4.66
Ensifera	3.39	1.50	0.79	14.54
Geometroidea	1.06	0.72	0.28	4.06
Heteroptera	1.69	0.25	0.39	7.25
Apidae (Honey bees)	23.75	7.01	9.51	59.30
Myrmeleontiformia	2.65	1.92	0.30	23.30
Noctuoidea	3.10	1.61	1.11	8.62
Parasitic wasps	5.67	2.13	1.91	16.84
Pyraloidea	1.32	0.85	0.37	4.71
Rhopalocera	4.25	1.47	1.35	13.38
Coleoptera: Scarabaeidae (beetles)	1.09	0.92	0.21	5.75
Coleoptera: ("other beetles")	2.07	1.13	0.70	6.10
Sphingidae	3.21	1.37	0.75	13.81
Tenebrionidae	0.53	0.38	0.06	4.66
Vespide (Wasps)	16.13	4.52	6.43	40.46
Apidae (Wild Bees)	3.93	1.99	1.45	10.65
Brachycera	1.55	0.39	0.64	3.78
Chironomidae	0.99	0.37	0.38	2.54
Apidae (Honey bees)	2.36	1.07	0.96	5.82
Noctuoidea	0.32	0.02	0.12	0.88
Vespidae (Parasitic wasps)	0.58	0.13	0.19	1.71
Rhopalocera	0.43	0.12	0.14	1.37
Vespidae (Wasps)	1.65	0.25	0.67	4.08
Apidae (Wild Bees)	0.40	0.12	0.15	1.08

LL—95% confidence interval lower limit, UL—95% confidence interval upper limit

With bait stations hung on walls at 1.8 m versus 1.0 m, stained non-targets were lower by 91.93%. However, Brachycera, wasps (Vespidae), and Chironomidae were still more predominant in the dye-marked group and had higher mean numbers compared to other insects.

## Discussion

The potential of Attractive Toxic Sugar Baits (ATSB) for control has been shown against populations of *Anopheles* vectors of malaria as well as several other mosquito species [2, 3, 5–7, 14, 15]. This study investigated ways to achieve adequate feeding by anophelines while minimizing exposure of the toxic bait to non-target organisms and using minimal investment of materials.

The ATSB system has several advantages. First, the proprietary SEBS membrane in this model bait station is easily penetrable by mosquito mouth parts so that they can land, probe and feed. Most insects that ingest sugars from exposed sources such as floral honeydew lack appropriate mouth parts to penetrate the membrane and feed. Second, because it can be used with several classes of insecticide [4–6] it can help alleviate the problem of resistance, and the flexibility of ATSB also allows use of low toxicity or arthropod-specific insecticides [2]. Last, it can be used as a spray or as a bait station depending on the needs of the user. The baits are used outdoors where the problem of reducing mosquitoes lies, in contrast to bed nets and indoor residual spraying. Finally, ATSB has inherently low primary and secondary toxicity towards non-target organisms [6, 8–10] and its effect is limited to sugar feeding insects that respond to the particular attractant of the baits. In previous studies, when sprayed on flowering vegetation where the local flora also exerts its attraction, aside from mosquitoes ATSB, without toxin, was fed upon by Diptera and generally it dye-marked up to 18% of the non-target insects on a daily basis [8, 11]. Dye-labelled Hymenoptera represented 15% of the caught population samples, Lepidoptera were 7%, and the remaining orders accounted for 5% 1.5% [8].

The advance from foliar ATSB spray to bait stations further reduced the number of non-target insects that fed on the bait [9], perhaps because bait stations, along with green vegetation, are not optical targets like flowers for sugar feeders while night active mosquitoes are drawn to the attractive scent [9]. In the current study, it was discovered that optimization of bait station placement could reduce the number of dye-marked specimens of non-target insect groups (Tables 5 and 6) for two reasons: (1) such locations were less in the course of their random flight paths and (2) stations could be moved out of the way of potential damage. Reducing the number of damaged stations could decrease feeding by non-target organisms. Feeding by mosquitoes was not affected by height but feeding by NTOs was higher among ATSBs at the lower height and therefore, the study indicates that bait stations should be placed higher on the walls of house (1.8 m; Table 2). Presentation of two bait stations with ASB marker per house led to a higher proportion of dye- marked mosquitoes than the presentation of one station. Placing three bait stations per house did not significantly increase the number of dye marked mosquitoes but it had the drawback of increasing the proportion of dye marked non-target insects (Tables 1, 5) as well as requiring more labour to hang and requiring more expenses. Regardless of the number of stations used, the number of stained non-targets was low; 57,902 non-target insect specimens were caught during the “number of stations” experiments, only 162 of which (0.3%) were dye-marked.

Even though height affects the number of stained non-targets, those numbers are ultimately very low. A total of 34,401 stained non-targets were captured, 446 (1.2%) had the dye of the lower stations while 32 (0.09%) had the dye of the higher stations. When bait stations were hung at 1.0 m, the majority of marked specimens in descending order were Brachycera (midges and other small flies), wasps (Vespidae), and honey bees (Apidae). At this height, day-to-day activities of residents caused minor damage that exposed the bait and allowed feeding from the stations by non-targeted organisms. This damage appears to be the major reason since the labelling of insects was diminished by about 90% when the ASB marker stations were at a height of 1.8 m. It is specifically noteworthy that honeybees were not marked by ASB stations at a height of 1.8 m (Tables 5 and 6).

The current study as with earlier observations [8–10, 14] shows that except for mosquitoes ATSB mainly affect Diptera (Brachycera), and to a lesser extent Hymenoptera (wasps, ants and honey bees) unless properly deployed. Much of the affected Diptera were filth flies that often rest on the walls of houses (observations of the authors). Altogether, the study shows that in these Malian villages ATSB stations placed at a height of 1.8 m maintain their effect on mosquitoes, and unlike lower bait station positions, have a negligible effect of non-target insects.

Optimization of ATSB deployment, either as a barrier spray [12] on green vegetation or even more so as bait stations, can further increase the specificity of ATSB and also increase the kill rate on mosquitoes. There is now evidence that the problem of affecting non-target insects can be minimised through optimising deployment. These principles of establishing efficient use and minimising NTO impact are simple enough to be transferable throughout Africa and globally though further testing in a number of different host countries would be needed to demonstrate this. Improvement of the protective membrane to resist damage and leakage will also most likely further reduce non-target impact. This demonstration in Mali of how bait station placement and configuration can minimize effects of ATSB on non-target insects provides a strong supportive case that ATSB stations can be used with confidence for the control of malaria parasite transmission by *Anopheles* mosquitoes with minimal harmful environmental impacts.

## Conclusion

In conclusion, this study demonstrates that optimization of bait stations, using such physical attributes as the number of stations and the height of these stations can maximize the number of mosquitoes attracted and killed while minimizing the unwanted effects on NTOs. In this case, 2 bait stations per house, hung at 1.8 m above ground limited damage to stations and subsequent invasion.

The minimal marking of non-target insects may be attributed to visual orientation of non-mosquito insects while mosquitoes, are mostly guided by olfactory cues.

## Data Availability

All data generated and analysed during this study are included in this published article.
